# LncRNA Sirt1-AS upregulates Sirt1 to attenuate aging related deep venous thrombosis

**DOI:** 10.18632/aging.202550

**Published:** 2021-02-26

**Authors:** Zhenkai Lou, Jinwen Zhu, Xing Li, Xingguo Li, Kaili Du, Bing Wang, Fan Zhang, Xinliang Zhang

**Affiliations:** 1 Department of Orthopedics, The First Affiliated Hospital of Kunming Medical University, Kunming 650032, Yunnan, China; 2 Department of Spine Surgery, Honghui Hospital, Xi’an Jiaotong University, Xi’an 710054, Shaanxi, China; 3 Department of Ultrasonography, The First Affiliated Hospital of Kunming Medical University, Kunming 650032, Yunnan, China

**Keywords:** long non-coding RNA Sirt1-AS, silent information regulator 1, deep vein thrombosis, senescence, human vascular endothelial cells

## Abstract

Aging is associated with the increased incidence of deep venous thrombosis (DVT), resulting in significant morbidity and mortality in the elderly, but the underlying mechanism is elusive. Silent information regulator 1 (Sirt1) is linked to the senescence, inflammation, oxidative stress and platelet adhesion of endothelial cells. Here we showed that DVT was associated with the senescence of endothelium and lower expression of Sirt1. Furthermore, Sirt1 could inhibit endothelial senescence and reduce the occurrence of DVT. Interestingly, we found antisense long non-coding RNA (lncRNA Sirt1-AS) upregulated Sirt1, decreased the expression of senescence and DVT associated biomarkers in human vascular endothelial cells (HUVECs). In addition, lncRNA Sirt1-AS overexpression alleviated DVT through upregulating Sirt1 and thereby inducing Foxo3a degradation. In conclusion, our findings demonstrate that lncRNA Sirt1-AS may be a potential new biomarker for DVT.

## INTRODUCTION

Deep vein thrombosis (DVT) of the lower extremities is a common complication in patients who suffer from trauma, and results in significant morbidity and mortality. Thrombus in circulating blood flow leads to pulmonary circulation and respiratory dysfunction, causing pulmonary embolism [1, 2]. However, the etiology and pathogenesis of DVT have not been completely understood. At present, DVT is mainly diagnosed by combining clinical manifestations with imaging, and lacks sensitive and specific biomarkers for early prediction, diagnosis and therapy. Recent studies revealed that DVT often develops with the increase of age [3]. Vascular endothelial cells (VECs) senescence and decreased anticoagulant and fibrinolytic activity play an important role in DVT development [4–6]. Therefore, it is necessary to explore senescence mechanism associated with DVT to develop effective biomarkers and potential therapeutic targets for DVT.

Silent information regulator 1 (Sirt1) is a NAD^+^ dependent lysine deacetylase activated in response to aging, energy metabolism, and various cellular stress [7–10]. It was reported that Sirt1 could be activated by resveratrol to extend the life of yeast by 70% [11]. Recent studies have shown that Sirt1 inhibits the development of many diseases, such as arterial thrombosis, atherosclerosis, myocardial ischemia-reperfusion injury and peripheral vascular pathology of diabetes [12–15], by regulating the senescence, inflammation, oxidative stress and platelet adhesion of endothelial cells [16–18].

Long non-coding RNAs (lncRNAs) are RNA molecules longer than 200 nucleotides without protein coding potential, and play important roles in diseases, such as cancer, cardiovascular disease, blood diseases, inflammation, and aging [19–21]. Based on chromosomal locations, lncRNAs are divided into several classifications: antisense, intronic, bidirectional, intergenic, and overlapping lncRNAs [22]. Antisense lncRNAs were defined as the lncRNAs transcribed from the opposite direction of the coding gene of their sense protein or a sense strand-derived RNA [23, 24]. Sirt1 antisense lncRNA (lncRNA Sirt1-AS) can bind and completely overlapping with 3’ untranslated region (3’-UTR) of Sirt1 mRNA and increase the stability of Sirt1 mRNA by forming a lncRNA-mRNA duplex and affect the expression of Sirt1 [25–27]. Accumulated evidence pointed out that the lncRNA Sirt1-AS involved in various diseases by increasing Sirt1 abundance [24, 28]. However, the effect of Sirt1 and mechanism of lncRNA Sirt1-AS on DVT has not been reported.

In the present study, we hypothesized that lncRNA Sirt1-AS upregulates Sirt1 expression to slow the aging process and attenuate aging-related DVT. We examined the expression of lncRNA Sirt1-AS and Sirt1 in thrombosis patients with different severity. Moreover, we investigated the role of lncRNA Sirt1-AS and Sirt1 in the progression of thrombosis in DVT mouse model. Furthermore, we determined the effects of lncRNA Sirt1-AS on the aging and thrombosis development of human umbilical vein endothelial cell (HUVECs).

## RESULTS

### LncRNA Sirt1-AS and Sirt1 expression decreased in patients with DVT

**Figure 1:**
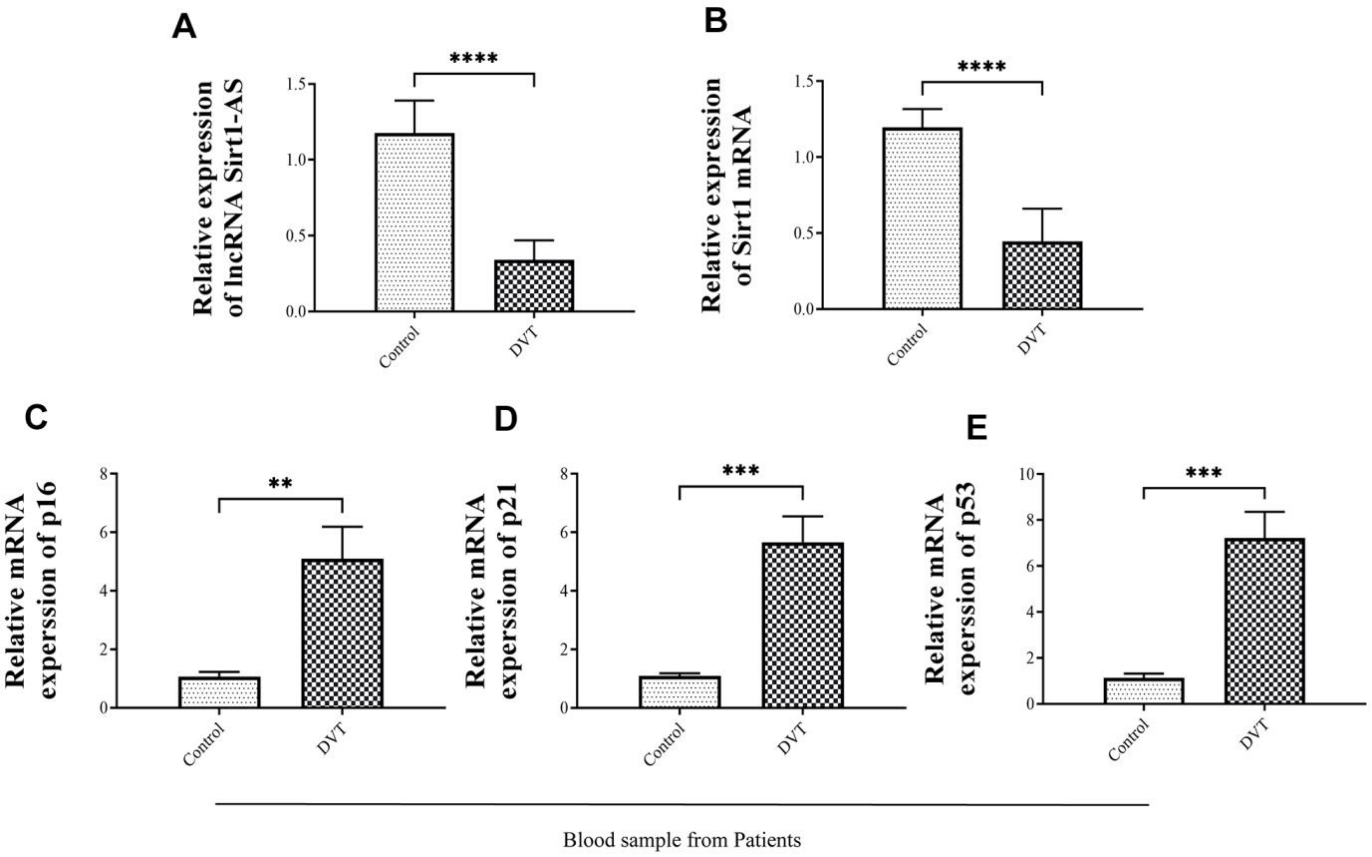
**The expression of senescence-related biomarkers, lncRNA Sirt1-AS and Sirt1 in thrombosis of different severity.** (**A**) Relative expression of lncRNA SIRT1-AS in thrombosis patients (**B**) Relative expression of mRNA Sirt1 in thrombosis patients. (**C**–**E**) Relative mRNA expressions of p16, p21 and p53 in thrombosis patients. Error bars represent SD. **, p < 0.01; ***, p < 0.001; ****, p < 0.0001.

First, we examined the relationship between the expression of lncRNA Sirt1-AS, Sirt1 and the severity of aging-related DVT. As shown in Figure 1A–1E, the expression of lncRNA Sirt1-AS and Sirt1 mRNA in the blood of thrombosis patients significantly decreased. In addition, the expression of cell senescence associated markers p16, p21 and p53 in the blood of thrombosis patients significantly increased.

### Sirt1 activation promoted DVT in SAMP-1 mice

**Figure 2:**
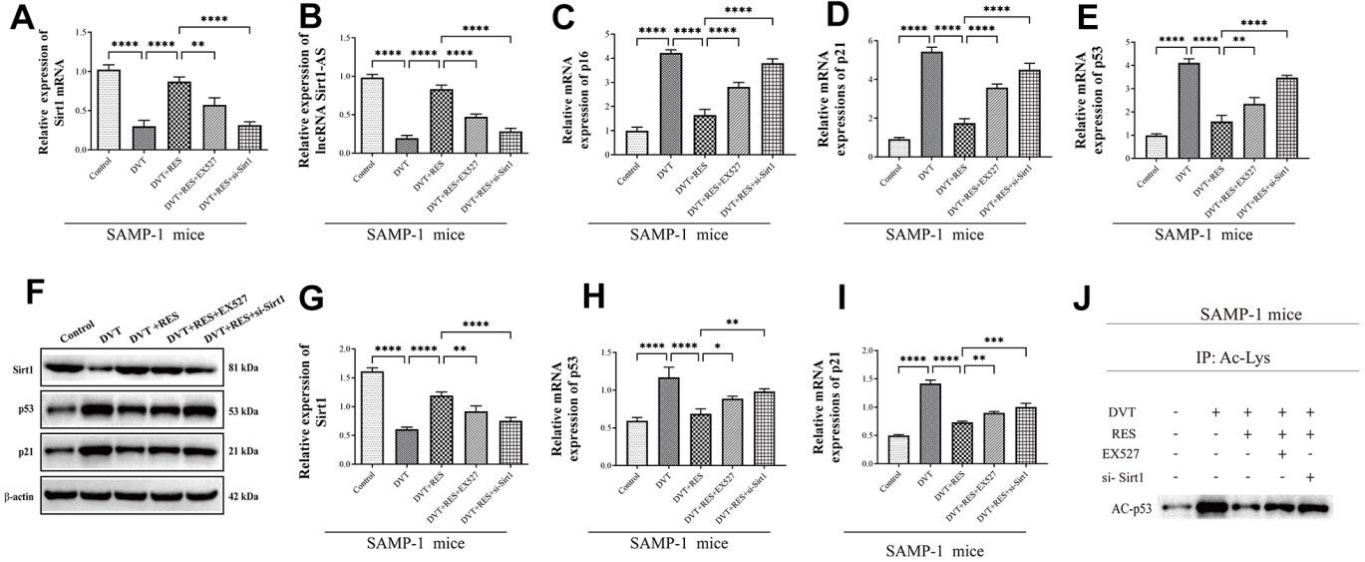
**LncRNA Sirt1-AS expression and Sirt1 activation in different groups of SAMP-1 mice.** (**A**) Relative mRNA expression of Sirt1. (**B**) Relative expression of lncRNA Sirt1-AS. (**C**–**E**) Relative mRNA expression of p16, p53 and p21. (**F**–**I**) Relative protein expression of Sirt1, p16, p53, and p21. (**J**) p53 acetylation was detected. Error bars represent SD. *, p< 0.05; **, p < 0.01; ***, p < 0.001; ****, p < 0.0001.

Our pervious study has proved that, resveratrol suppressed the expression of sirt1 and thereby decreased the expression of thrombosis-related markers P-selectin, P-selectin glycoprotein ligand 1 (PSGL-1), and Von Willebrand factor (VWF) in HUVEC [29], We therefore used SAMP-1 mice to establish DVT model and to further investigated the effect of Srit1 on DVT. Resveratrol promoted the activation of Srit1, while inhibitor EX527 and si-Sirt1 reversed the promotion (Figure 2A, 2G). The expression of lncRNA Sirt1-AS increased when Sirt1 was activated by resveratrol, but decreased when Sirt1 was inhibited by inhibitor EX527 and si-Sirt1 (Figure 2B). Resveratrol decreased the expression of p16, p21 and p53 compared to DVT group (Figure 2C–2E, 2H, 2I), while EX527 and si-Sirt1 significantly increased the expression of p16, p21 and p53. Since Sirt1 can inactivate p53 by deacetylating it, the expression of acetylated p53 increased in DVT model and decreased significantly when resveratrol was applied, but the effect was reversed by EX527 and si-Sirt1 (p < 0.05, Figure 2J).

**Figure 3:**
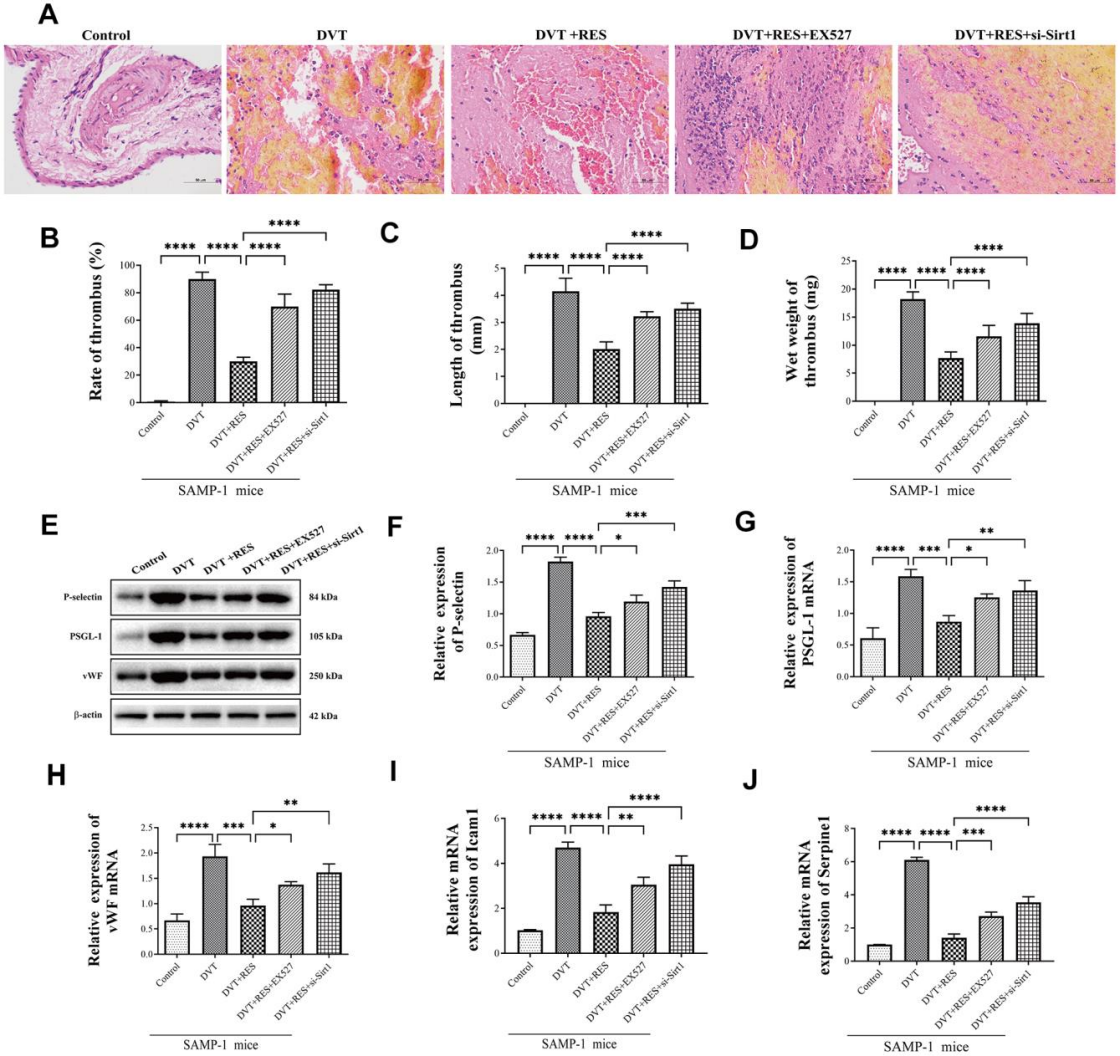
**Sirt1 activation suppresses DVT in SAMP-1 mice.** (**A**) HE staining of endothelia in different groups of SAMP-1 mice (40*). (**B**–**D**) mRNA and protein expression of P-selectin, PSGL-1 and vWF in different groups of SAMP-1 mice. (**E**–**H**) The rate of thrombus, length and wet weight of thrombus in different groups of SAMP-1 mice. (**I**, **J**) mRNA expression of senescence associated genes Icam1 and Serpine1. Error bars represent SD. *, p< 0.05; **, p < 0.01; ***, p < 0.001; ****, p < 0.0001.

Next, HE staining was performed to evaluate endothelial aging. Compared to DVT model, the fibrin content in the thrombus was reduced when resveratrol was applied but the results were reversed by EX527 and si-Sirt1 (Figure 3A). Coincidently, compared with control group, the rate, length and wet weight of thrombus in model group significantly increased in DVT model. (Figure 3B–3D). When treated with resveratrol, the rate, length and wet weight of thrombus were remarkably reduced than DVT model, while these effects were inhibited by EX527 and si-Sirt1. Moreover, mRNA and protein expression of thrombosis-related markers P-selectin, PSGL-1, and VWF significantly increased in DVT model compared to control (Figure 3E–3H). Resveratrol significantly reduced mRNA and protein expression of P-selectin, PSGL-1 and vWF in DVT model, but the effects were reversed by EX527 and si-Sirt1. Compared with control group, the mRNA expression of senescence-associated genes Icam1 and Serpine1 increased in DVT model, but decreased after resveratrol was applied, and the effects were reversed by Sirt1 inhibitor EX527 and si-Sirt1 (Figure 3I, 3J). The results showed that activating Sirt1 could decrease the incidence of DVT.

### Sirt1 is a target of lncRNA Sirt1-AS

**Figure 4:**
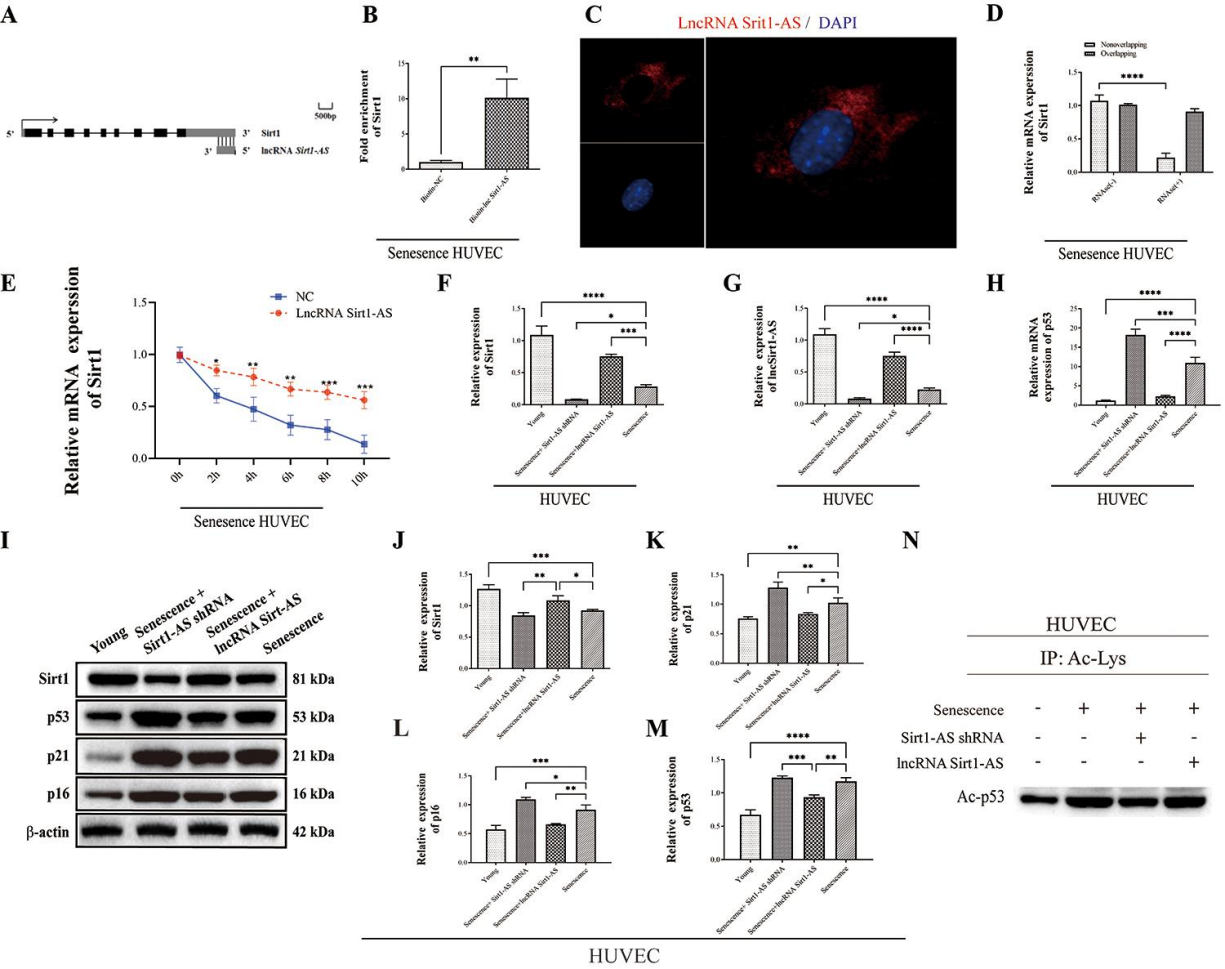
**Sirt1 is a target of lncRNA Sirt1-AS.** (**A**)Structure of human Sirt1 gene and location of lncRNA Sirt1-AS. (**B**) RNA-RNA pull-down assay. (**C**) Fish assay was performed to observe the location of LncRNA Sirt1-AS in senescence HUVECS. (**D**) ribonuclease protection assay was performed to detect the duplex formation of lncRNA Sirt1-AS and Sirt1. (**E**) Actinomycin D assay was used to detect the stability of Sirt1 mRNA. (**F**) Relative expression of Sirt1. (**G**) Relative mRNA expression of lncRNA Sirt1-AS. (**H**) Relative mRNA expression of p53. (**I**–**M**) Relative protein expression of Sirt1, p16, p53 and p21. (**N**) p53 acetylation was detected. Error bars represent SD.*, p< 0.05; **, p < 0.01; ***, p < 0.001; ****, p < 0.0001.

LncRNA Sirt1-AS is a natural antisense transcript (NAT) RNA (758 bp) located at the same gene locus as Sirt1 (3’ untranslated region (3’-UTR)), with a tail-to-tail fully overlapping complementary sequence by base complementary pairing principle [24] (Figure 4A, https://mafft.cbrc.jp/alignment/server/spool/_ho.20112409466225v6zV7Juc8Oe9gDyBltofSlsfnormal.html). To reveal the relationship between lncRNA Sirt1-As and Sirt1, we performed RNA pulldown assay, the result verified the competitive binding of lncRNA Sirt1-AS and Sirt1 in senescence HUVECs (Figure 4B). Also, result of RNA Fluorescence *in Situ* Hybridization showed that lncRNA Sirt1-AS was located in the cytoplasm of senescence HUVECs (Figure 4C). The ribonuclease protection assay (RPA) was performed to detect the duplex formation of lncRNA Sirt1-AS and Sirt1. As shown in Figure 4D, the overlapping part of both transcripts was protected from degradation, indicating the formation of duplex. Also, actinomycin D was used to suppress transcription and loss of Sirt1 mRNA expression was detected via qPCR. Obviously, lncRNA Sirt1-AS prolonged the half-life of Sirt1 mRNA, which suggested that lncRNA Sirt1-AS enhanced the stability of Sirt1 mRNA (Figure 4E).

**Figure 5:**
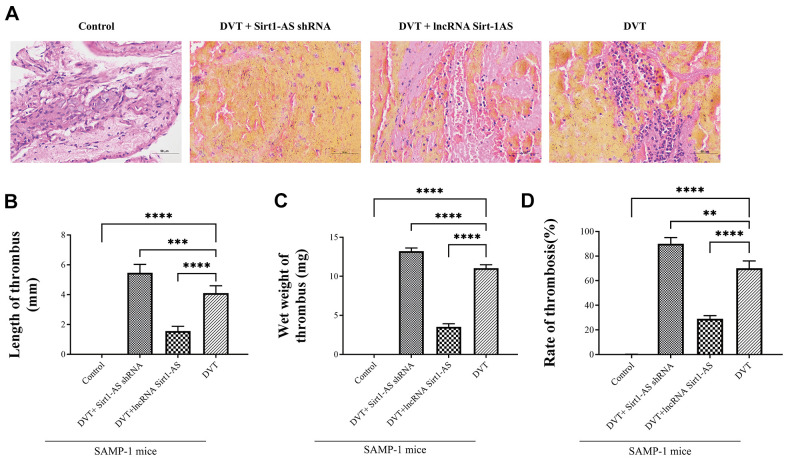
**LncRNA Sirt1-AS upregulates Sirt1 and alleviates DVT.** (**A**) HE staining of endothelia of SAMP-1 mice. (40*). (**B**–**D**) The rate of thrombus, length and wet weight of thrombus of SAMP-1 mice. Error bars represent SD. **, p < 0.01; ***, p < 0.001; ****, p < 0.0001.

To confirm that Sirt1 is the target of lncRNA Sirt1-AS in the process of DVT, we detected the expression of lncRNA Sirt1-AS after the transfection of lncRNA Sirt1-AS shRNA or pcDNA vector (lncRNA Sirt1-AS) in normal or replicative senescence HUVECs. The expression of lncRNA Sirt1-AS was decreased in senescence HUVECs and further decreased after Sirt1-AS shRNA was transfected, but elevated after lncRNA Sirt1-AS was transfected. Compared to senescence HUVECs, the mRNA and protein expression of Sirt1 increased when lncRNA Sirt1-AS was overexpressed (Figure 4F, 4G, 4J). In addition, expression of p21, p16, p53 and acetylated p53 all increased, indicating the activation of Sirt1 by lncRNA Sirt1-AS (Figure 4H, 4I–4N). HE staining showed remarkably decreased fibrin content after lncRNA Sirt1-AS transfection and increased fibrin content after Sirt1-AS shRNA transfection (Figure 5A). Compared with DVT group, the rate, length and wet weight of thrombus in lncRNA Sirt1-AS group were significantly decreased while Sirt1-AS shRNA increased the rate length and wet weight of thrombus (Figure 5B–5D). These results indicate that the overexpression of lncRNA Sirt1-AS could attenuate the development of DVT via targeting Sirt1.

### LncRNA Sirt1-AS upregulated Sirt1 to delay aging and alleviate DVT

**Figure 6:**
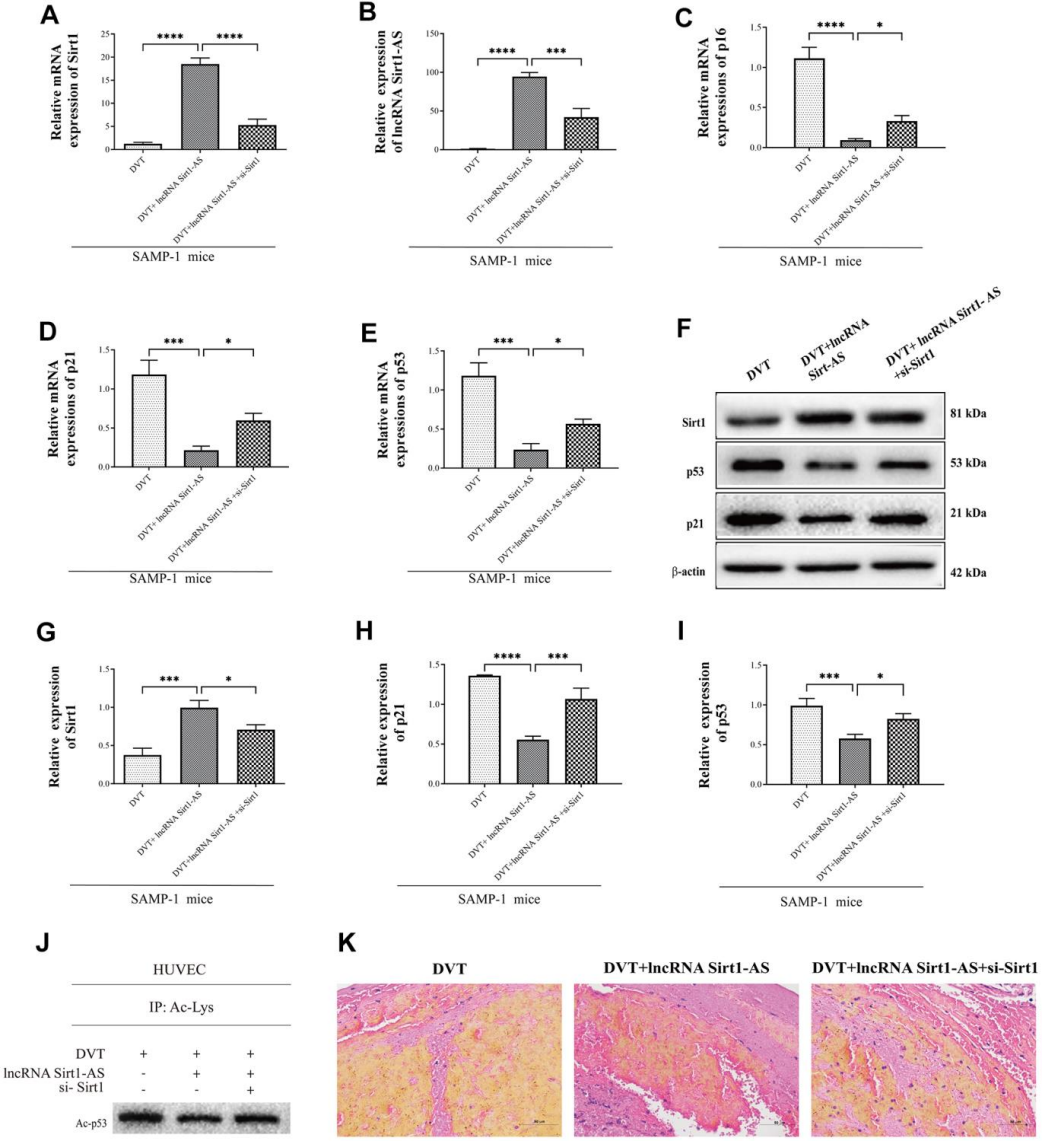
**Sirt1 is upregulated by lncRNA Sirt1-AS overexpression.** (**A**, **B**) Expression of Sirt1 mRNA and lncRNA Sirt1-AS. (**C**–**E**) Relative mRNA expression of p16, p53 and p21. (**F**–**I**) Protein levels of Sirt1, p16, p53 and p21. (**J**) p53 acetylation was detected. (**K**) HE staining of endothelia of SAMP-1 mice. (40*). Error bars represent SD. *, p< 0.05; ***, p < 0.001; ****, p < 0.0001.

**Figure 7:**
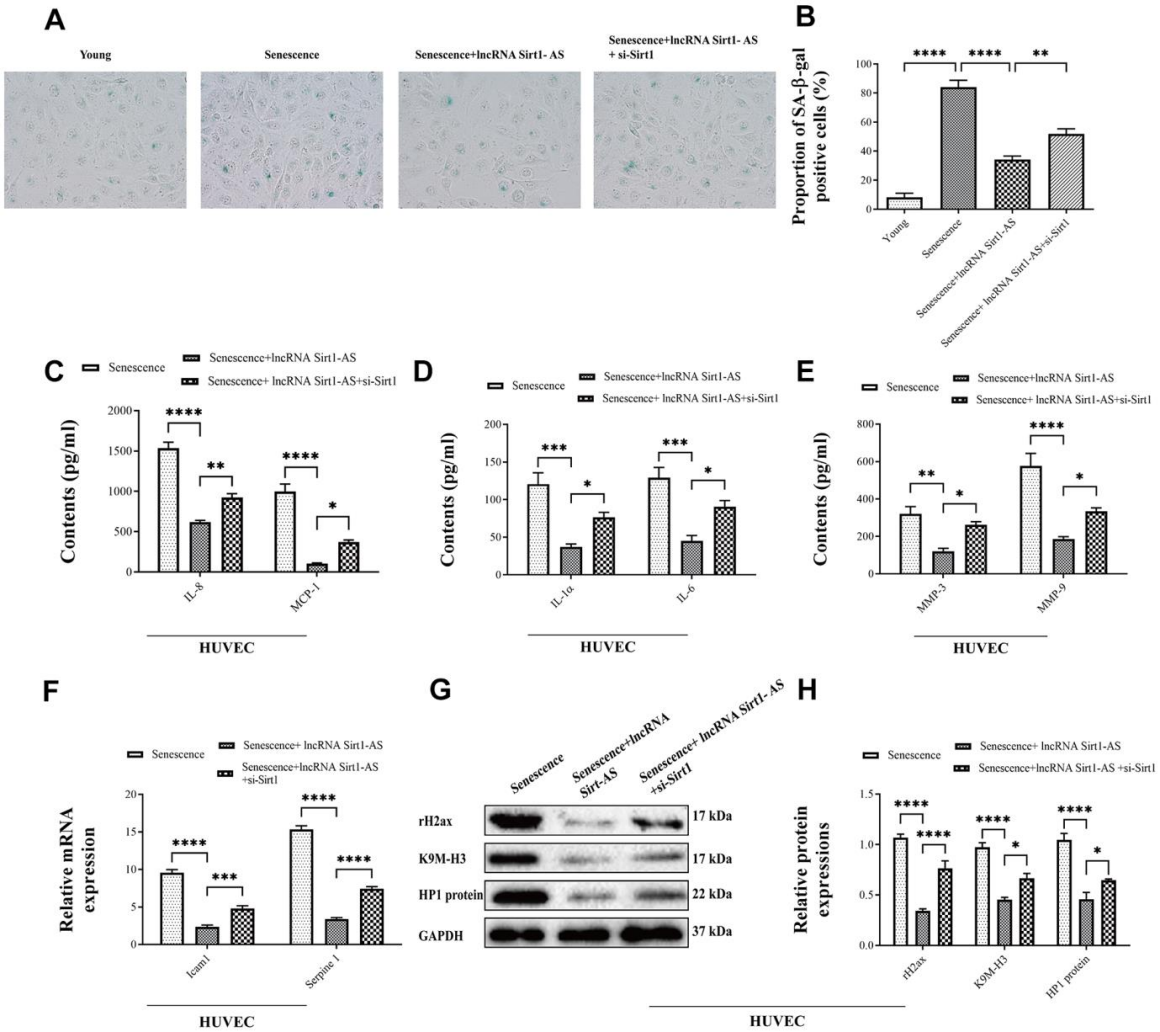
**Effects of lncRNA Sirt1-AS overexpression on HUVECs senescence.** (**A**, **B**) The proportion of SA-β-Gal positive cells. (**C**–**E**) The contents of IL-8, MCP-1, IL-1α, IL-6, MMP-3 and MMP-9 after the transfection of lncRNA Sirt1-AS and treatment of Sirt1 inhibitor. (**F**) mRNA expression of senescence associated genes Icam1 and Serpine1. (**G**, **H**) The expression of senescence-associated markers rH2, K9M-H3, and HP1. Error bars represent SD. *, p< 0.05; **, p < 0.01; ***, p < 0.001; ****, p < 0.0001.

Compared with DVT model, mRNA expression of Sirt1 and Sirt1-AS increased after pcDNA vector was transfected, but was reversed after si-Sirt1 was applied. Same trend was found in the activation of Sirt1 and the expression of p21 and p53 and acetylated p53 (Figure 6A–6J). The result of HE staining showed that, the fibrin content in the thrombus was reduced when the expression of lncRNA Sort1-AS was increased whereas the result reversed when si-Sirt1 was transfected (Figure 6K). Compared with the young HUVECs, the proportion of SA-β-gal positive cells in senescence HUVECs was significantly increased, and remarkably reduced by lncRNA Sirt1-AS shRNA stimulation (Figure 7A, 7B). Meanwhile, the effects of lncRNA Sirt1-AS on SA-β-gal positive cells was inhibited by si-Sirt1. As shown in Figure 7C–7E, IL-8, MCP-1, IL-1α, IL-6, MMP-3 and MMP-9 levels were significantly reduced after the transfection of lncRNA Sirt1-AS, but could be reversed by si-Sirt1. In addition, compared with the senescence HUVECs, the expression of aging-related genes in lncRNA Sirt1-AS overexpression group were significantly decreased, including lcam1 and serpine1 (Figure 7F). Similarly, the effects of lncRNA Sirt1-AS were suppressed by si-Sirt1. Moreover, lncRNA Sirt1-AS overexpression markedly decreased rH2ax, K9M-H3and HP1 expression (Figure 7G, 7H).

**Figure 8:**
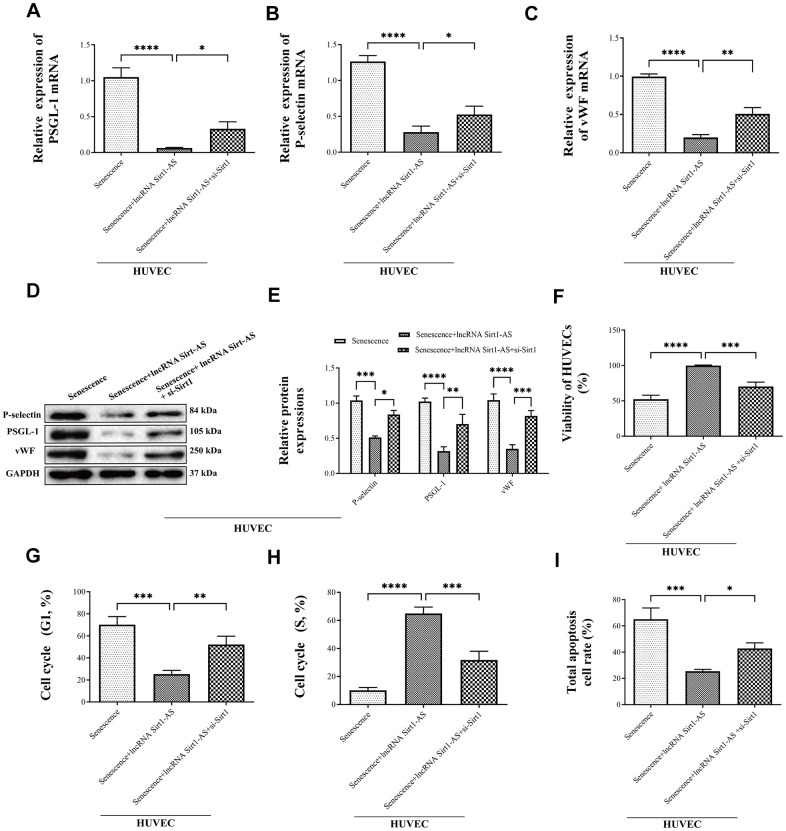
**Effects of lncRNA Sirt1-AS overexpression on mRNA and protein expression of P-selectin, PSGL-1 and vWF in different treatment of SAMP-1 mice.** (**A**–**E**) and the HUVECs viability (**F**), cycle (**G** in G1 phase, **H** in S phase) and apoptosis (**I**). Error bars represent SD. *, p< 0.05; **, p < 0.01; ***, p < 0.001; ****, p < 0.0001.

Furthermore, compared with the senescence HUVECs, mRNA and protein expression of thrombosis-associated markers P-selectin, PSGL-1 and vWF in lncRNA Sirt1-AS overexpression group were significantly reduced (Figure 8A–8E). However, si-Sirt1 increased mRNA and protein expression of P-selectin, PSGL-1 and vWF. These results demonstrated that DVT may be alleviated by lncRNA Sirt1-AS overexpression, but the effects of lncRNA Sirt1-AS could be weakened when Sirt1 activation was inhibited.

We then explored the effects of lncRNA Sirt1-AS on the cell viability, cycle and apoptosis. LncRNA Sirt1-AS overexpression increased the viability of HUVECs but si-Sirt1 significantly decreased cell viability (Figure 8F). Compared to the senescence HUVECs, the number of cells in the G1 phase was remarkably decreased and the number of cells in the S phase was increased when pcDNA vector was transfected, but the effects of lncRNA Sirt1-AS were inhibited by si-Sirt1 (Figure 8G, 8H). Furthermore, lncRNA Sirt1-AS treated cells had decreased apoptosis, which was abolished by si-Sirt1 (Figure 8I).

### LncRNA Sirt1-AS regulated DVT through FOXO3a ubiquitination and degradation

**Figure 9:**
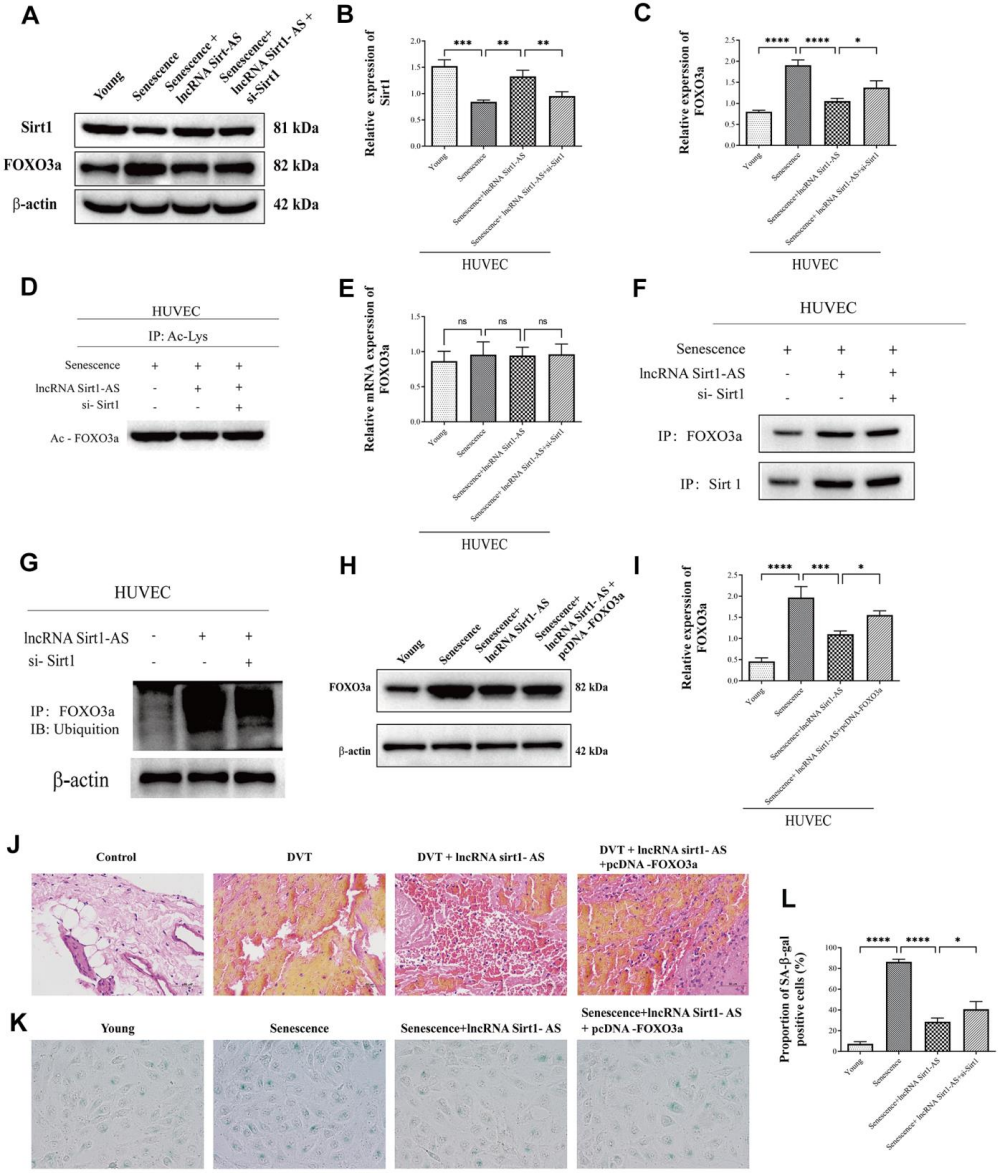
**LncRNA Sirt1-AS regulated DVT through FOXO3a ubiquitination and degradation** (**A**–**D**) Protein expression of Sirt1, FOXO3a and its acetylation were detected. (**E**) Relative mRNA expression of FOXO3a. (**F**) Co-IP was performed to detect the interaction of Sirt1 and FOXO3a. (**G**) Western blot was used to detect the FOXO3a ubiquitination level. (**H**, **I**) Western blot was used to detect the protein expression of FOXO3a after pcDNA3.1-FOXO3a transfection. (**J**) HE staining of endothelia of SAMP-1 mice. (40*) (**K**, **L**) proportion of SA-β-Gal positive cells. Error bars represent SD. *, p< 0.05; **, p < 0.01; ***, p < 0.001; ****, p < 0.0001.

Prevailing evidence showed that, Sirt1 was bind to FOXO3a, reduce its acetylation level and increase its ubiquitination level, and therefore decreased the expression of FOXO3a [30, 31]. We therefore assume LncRNA Sirt1-AS regulated DVT through FOXO3a ubiquitination and degradation. We thus determined the level of FOXO3a ubiquitination and degradation and how this exerted on DVT generation. The results of western blot showed that, the expression of Sirt1 was increased in senescence HUVECs compared to young HUVECs, while the expression was increased after pcDNA vector was transfected. The transfection of si-Sirt1 reversed the result. Also, the expression of FOXO3a and its acetylation was increased in senescence HUVECs, while the expression was decreased after pcDNA vector was transfected. The transfection of si-Sirt1 reversed the result. (Figure 9A–9D) Which indicated that the expression of FOXO3a and its acetylation was regulated by Sirt1, which is stabilized by lncRNA Sirt1-AS, the overexpression of sirt1 deacetylated FOXO3a. Also, qPCR was performed to detect the mRNA expression of FOXO3a. No significant different was found among groups (Figure 9E). Which confirming that Sirt1 reduced the protein expression of FOXO3a through FOXO3a post-transcriptional regulation. Furthermore, the interaction of Sirt1 and FOXO3a was verified via Co-IP assay (Figure 9F). In senescence HUVECs, the immune complex was increased after pcDNA vector was transfected. The transfection of si-Sirt1 reversed the result. Similarly, the level of ubiquitination was detected via western blot (Figure 9G). In senescence HUVECs, the ubiquitination level of FOXO3a was increased after pcDNA vector was transfected. The transfection of si-Sirt1 reversed the result. The result showed that Sirt1 deacetylated FOXO3a and thereby promoted its ubiquitination and degradation.

To evaluate the effect of FOXO3a protein expression change on DVT generation, pcDNA3.1-FOXO3a was transfected. Western blot was performed to detect the protein expression of FOXO3a (Figure 9H, 9I). The result showed that, the expression of FOXO3a was decreased after pcDNA vector was transfected, while the transfection of pcDNA3.1-FOXO3a reversed the result. Which suggested the successful transfection. HE staining showed that pcDNA-FOXO3a increased fibrin content, indicating the aggression of DVT (Figure 9J). Coincidently, the result of SA-β-gel assay (Figure 9K, 9L) showed that pcDNA-FOXO3a aggregated senescence of HUVECs, indicating the aggregation of DVT. These results indicate that Sirt1-AS inhibits DVT through FOXO3a ubiquitination and degradation.

## DISCUSSION

DVT involves the interaction of vascular endothelial cells, platelets and coagulation-related proteins. Deep vein thrombosis is asymptomatic and easily neglected in its early stages. [32, 33]. Aging is generally considered as a crucial cardiovascular risk factor [34]. Rate of vein thromboembolism is found dramatically increased as the population ages. To be specific, with 1 per 10,000 events in the population less than 40 years old, while 1 per 1000 in the population over 75 years old [35]. The structural change of vasculature is observed in venous system. With the increasing of age, venous valve appears thickened at the base and thinner at the end of the valve leaflet, as well as supported by less organized collagen [36]. Which directly induces the dysfunction of endothelium and thus increases the risk of thrombosis. Endothelial cells are associated with the resolution of fibrin as long as thrombus formation was started [37]. Senescent HUVECs loss their function when their irreversible morphological changes happened. To be specific, the proliferation potential was deprived, cell size was increased, as well as cell nuclei becomes irregular [38]. However, the molecular mechanism of aging and DVT is yet unclear. In present study, we showed that lncRNA Sirt1-AS upregulated Sirt1 expression to slow the aging process and attenuate aging-related DVT.

Sirt1 plays multiple roles in longevity, apoptosis, DNA repair, inflammation, and mitochondrial regulation [39, 40]. Tumor suppressor protein 53 is associated with replicative senescence [41]. Acetylation of nuclear p53 enhances its stabilization, hence promotes the transcription of genes in the cascades of antioxidant, oxidant and/or pro-apoptosis, therefore accelerate aging [42]. As a downstream factor of Sirt1, p53 was deacetylated by Sirt1 [43] and decreased transcriptional activity induced by p53. The expression of downstream protein such as p21 and p16 is also reduced [44, 45]. Hidetaka el at [46] found that suppression of Sirt1 increased the level of p53 acetylation and therefore induced senescence -like phenotype in human endothelial cells. Christina et al [47] also proved that, the increase expression of p53 promotes endothelial senescence, while the activation of Sirt1 can inhibit the activation of p53/p21 pathway and therefore suppressing endothelial cell senescence. Coincident with our results, the expression and activation of Sirt1 decreased in DVT patients. Moreover, the activation of Sirt1 decreased the expression of p53 and its acetylation, thereby suppressed the development of endothelial aging and alleviated the formation of thrombus, but these results were reversed by Sirt1 inhibition.

Sirt1-AS is an antisense lncRNA of Sirt1, and the expression and the activation of Sirt1 decreased when the expression of lncRNA Sirt1-AS was suppressed, validating that Sirt1-AS is an antisense lncRNA of Sirt1. Wang et al. [25] provided evidence that Sirt1 AS lncRNA interacted with Sirt1 3’ UTR and rescued Sirt1 transcriptional suppression by competing with miR-34a. Li et al. [24] found that Sirt1 antisense lncRNA can bind the Sirt1 30-untranslated region, enhancing the stability of Sirt1 and increasing Sirt1 abundance at both mRNA and protein levels. We found that the overexpression of lncRNA Sirt1-AS increase the expression of Sirt1, and suppressed the development of endothelial aging and alleviated the formation of thrombus. Also, the overexpression of lncRNA Sirt1-AS reversed the decrease of the HUVEC viability and increase of the HUVECs apoptosis, as well as the cell cycle arrested by senescence.

Forkhead box O (FOXO) 3a is transcription factor, of which the transcription activity can be regulated by post-translational modifications such as phosphorylation and acetylation [48]. As Sirt1 is a deacetylase that both ubiquitination and acetylation modification take placed on the same group of lysine residues, Sirt1 can inhibit FOXO3a activation by reduce its acetylation level and increase its ubiquitination level, and finally induce its degradation [35, 49, 50]. Conversely, the expression of Sirt1 is mediated by FOXO3a. The promoter of Sirt1 contains two p53 binding elements. FOXO3a binds to p53 to form a protein complex, which drives the expression of Sirt1 [51]. Ham et al. [48] recently found the defect of Sirt1/FOXO3a axis induced reactive oxygen species (ROS) accumulation and finally aggravated the cell senescence. Similarly, we found Sirt1 can decrease the expression of FOXO3a by decrease its acetylation and increased its ubiquitination level in senescence HUVECs. Also, we proved that the overexpression of FOXO3a can reversed the mitigation effect of lncRNA Sirt1-AS on HUVECs aging and DVT generation.

In conclusion, Sirt1-AS is an antisense lncRNA of Sirt1, and the development of DVT is associated with endothelial aging, along with the reduction of lncRNA Sirt1-AS and Sirt1 expression. Sirt1 decreases the incidence and generation of aging-related DVT by the delay of aging. LncRNA Sirt1-AS decreases the incidence of aging-related DVT by increasing viability and proliferation of HUVECs, as well as decreasing the apoptosis of HUVECs. LncRNA Sirt1-AS alleviates DVT via regulating Sirt1/Foxo3a axis.

## MATERIALS AND METHODS

### Ethics statement

All animal experiments were approved by Animal Ethics and Welfare Committee and performed in accordance with the "Guidelines for *in vivo* experimental research reports in animal experiments" (ARRIVE Guidelines).

Clinical protocols were approved by the Ethics Review Committee of First Affiliated Hospital of Kunming Medical University. Written informed consent was obtained from all patients or their legal guardian.

### Clinical samples

Blood samples were obtained from the patients who suffered from thromboembolism in saphenous vein. A total of 50 patients (21 males and 29 females, mean age: 47.37 ± 11.72 years old) were enrolled in the present study. All patients were diagnosed by duplex scan, along with healthy controls. Exclusion criteria included participants younger than 18 years, unwilling to consent, actively pregnant, or on immunosuppressant or anticoagulant therapy. Patients with isolated calf venous thrombosis were also excluded. Clinicopathological features of control volunteers and patients with DVT were shown on Table 1.

**Table 1:** Clinical characteristics of participants.

	**Control**		**DVT**
	**n = 50**		**n = 50**
Age (years)	45.64±11.65		47.37±11.72
Gender (Male/Female)	24/26		21/29
BMI (kg/m2)	23.21±3.60		24.64±3.91
HDL (mmol/L)	1.28±0.28		1.19±0.33
LDL (mmol/L)	2.90±0.64		2.72±0.69
**DVT**			
Proximal	/		23
Distal	/		27

### HUVECs culture and transfection

Human umbilical vein endothelial cells (HUVECs) were obtained from ATCC (VA, USA, cat. No. PCS-100-010). Cells were cultured in Dulbecco's modified eagle medium (DMEM) supplemented with 10% FBS, 100 U/ml penicillin and 100 μg/ml streptomycin at 37° C with 5% CO_2_ in a humidified atmosphere. Replicative senescence of HUVECs was induced by serial passage of HUVECs [52, 53]. Briefly, cells were harvested and serial passage were performed in the interval of 5-9 days (seed density: 5000 cells/cm^2^). The passage of cells in control group (“young cell”) were selected when the less than 10% of the population expressed senescence-associated β-galactosidase (SA-β-gal). In contrast, senescence cells (senescence group) were selected under the following conditions [53]: 1) cells did not increase in numbers during 4 weeks; 2) cells exhibited altered morphology; 3) the positive rate of SA-β-gal staining is more than 70% of population.

Full-length lncRNA Sirt1-AS was subcloned into pcDNA vector (Ke Lei Biological Technology Co., Ltd). Cells were transfected with shSirt1-AS vector (Genechem Shanghai, China), empty pcDNA vector, empty pBABE vector, pcDNA vector (lncRNA Sirt1-AS), pBABE vector (si-Sirt1) and pcDNA vector plus pcDNA-FOXO3a, or with si-Sirt1 (Santa Cruz Biotechnology, Inc.) by using Lipofectamine 2000 (Invitrogen, Carlsbad, CA, USA).

### CCk-8 assay and flow cytometry

Viability of HUVECs were evaluated using Cell Counting Kit-8 (CCK-8, Dongren Chemical Technology co., Ltd, Shanghai, China), according to the manufacturer’s instructions. Cells were incubated with CCK-8 reagent for 1 h, and absorbance at 450 nm was measured with a microplate reader (Tecan, Männedorf, Switzerland).

For flow cytometry, cell was fixed with pre-cooled 70% alcohol for 24 h, washed with PBS and then incubated with propidium iodide (PI) (Dongren Chemical Technology co., Ltd, Shanghai, China) under dark condition at 4° C. Cell cycle stages were detected by a flow cytometer (Partec GmbH, CyFlow Space). Cell apoptosis was detected using the Annexin V-FITC/PI staining. 100 μl of 1×10^6^ cells/ml cell suspension was added into 5 μl of Annexin V-FITC (Partec GmbH, CyFlow Space) and 10 μl of PI, and the culture was incubated at room temperature for 15 min and washed twice with PBS. Cell apoptosis was analyzed using a flow cytometer at an excitation wavelength of 488 nm.

### Senescence-associated β-galactosidase (SA-β-gal) staining

Cells were stained by using the senescence-associated β-galactosidase (SA-β-gal, Solarbio, China) detection kit. SA-β-gal positive cells were stained in blue. Five fields of view were randomly selected under an optical microscope and senescence index (SI) was calculated using the following formula: SI= positive cells/total cells×100%.

### RNA extraction and sequencing

Total RNA was extracted from patient blood and mice endothelial samples using a TRIzol reagent (Lifetech). 1.5 μg RNA was used to construct library using a NEB Next Ultra small RNA Sample Library Prep Kit (Fermentas), and then sequenced on an Illumina HiSeq2500 platform (Fermentas). The transcriptome sequencing of mRNA and lncRNA was performed on an Illumina gene analyzer (Invitrogen). The data were deposited at National Center for Biotechnology Information (NCBI) Sequence Read Archive (SRA) database under accession number: SRP092509. Quality control of the sequencing data was performed to identify the clean reads using the FASTX-toolkit. The obtained clean reads were aligned to the human reference genome hg19 using TopHat software [54].

### RNA fluorescence *in situ* hybridization (FISH)

RNA fluorescence *in situ* hybridization was performed to observe the location of LncRNA Sirt1-AS as described previously [24]. Senescence HUVECs was fixed and washed, then permeabilized in 0.2% Triton X-100 PBS, hybridized with a hybridization solution, and incubated overnight at 37° C with a labeled lncRNA Sirt1-AS probe (Sangon, China). Cell then incubated with a mouse antibody to antidigoxin conjugated with alkaline phosphatase (Sigma, China) after washed. Then cells were incubated with 4’, 6-diamidino-2-phenylindole (Best-bio, Shanghai, China). The image was acquired by laser scanning confocal microscopy (LSM710, Carl Zeiss, Germany).

### Ribonuclease protection assay (RPA) and actinomycin D assay

Ribonuclease Protection Assay (RPA) was performed to detect the sense-antisense RNA duplex in senescence HUVECs [24]. In brief, the oligonucleotides used for RPA was synthesis as described sequences. Cytoplasmic RNA was orderly digested by DNaseI and RPA-grade RNase A (Applied Biosystems, CA) to remove all genomic DNA contamination and single-stranded RNAs. After cDNA synthesis, RT-qPCR was performed to detected the mRNA expression of Sirt1 as described.

Also, Actinomycin D assay was used to detect the stability of Sirt1 mRNA [24]. In brief, senescence HUVECs was treated with 2 μg/mL to block transcription and cells were harvested at 0, 2, 4, 6, 8, 10 h after treatment respectively. The total RNA was extracted and residual mRNAs of Sirt1 was detected via RT-qPCR. GAPDH was used as loading control.

### RNA pulldown assay

The 3’end biotinylated LncRNA Sirt1-AS probe or control probe (Ribio, China) were transfected into HUVECs at a final concentration of 50 nM. Lysis buffer (Invitrogen, USA) and complete protease inhibitor cocktail (Roche Applied Science, IN) were added into the cell pellets. The biotin-coupled RNA complex was pulled down by incubating the cell lysates with streptavidin-coated magnetic beads (Life Technologies). The abundance of Sirt1 was evaluated by qPT-PCR [55].

### DVT model in SAMP-1 mice

12-month- old SAMP-1 mice were purchased from Auno Biotechnology Co., Ltd., China and kept at room temperature (20~25° C) and relative humidity (60~70%) with free access to food and water. After inhalation anesthesia, a 1.5-2 cm incision along the midline of the abdomen was taken, and then cut the skin, subcutaneous tissue, abdominal muscles, peritoneum to abdominal cavity respectively, and contents of the abdominal intestine, omentum and other contents out of the abdominal cavity was peeled, which then covered with hot saline gauze. Then, the inferior vena cava (IVC) was fully exposed using the ophthalmic eyelash hook and peeled with a surgical microscopic hook. The IVC and all visible side branches (usually 2 or 3) were ligated with nonreactive 6-0 silk sutures. Mice were randomly divided into following groups (n = 10): sham group (control group, mice without IVC), IVC treatment group (DVT group), IVC + resveratrol group (DVT+RES, IVC mice were administrated by resveratrol (20 mg/kg) for 14 days), IVC + resveratrol plus EX527 group (DVT+RES+EX527, IVC mice were administrated by resveratrol (20 mg/kg) and EX527 (1 μg/kg) for 14 days), IVC + resveratrol plus si-Sirt1 group (DVT+RES+ si-Sirt1, IVC mice were administrated by resveratrol (20 mg/kg) for 14 days and si-Sirt1 was intraperitoneal injected for continues 7 days), IVC+ pcDNA vector (DVT+ lncRNA Sirt1-AS, pcDNA vector was intraperitoneal injected for continues 7 days), IVC+Sirt1-AS shRNA (DVT+ Sirt1-AS shRNA, shSirt1-AS vector was intraperitoneal injected for continues 7 days), IVC+ pcDNA vector+si-Sirt1 (DVT+ lncRNA Sirt1-AS+si-Sirt1, pcDNA vector and si-Sirt1 were intraperitoneal injected for continues 7 days), IVC + pcDNA vector+ pcDNA-FOXO3a (DVT+ lncRNA Sirt1-AS+ pcDNA-FOXO3a, pcDNA vectors were intraperitoneal injected for continues 7 days). Resveratrol and EX527 were dissolved in dimethyl sulfoxide (DMSO) and phosphate buffer solution (PBS) (DMSO: PBS = 1:1) and stored at 4° C. The IVC and the associated thrombus of each group were removed, weighed, and measured for thrombus rate and length after model establishment.

### Hematoxylin and eosin (HE) staining

Endothelial samples were fixed in 4% (w/v) paraformaldehyde and embedded in paraffin. The 4-micron thick slices were deparaffinized by xylene and hydration, and then stained with hematoxylin and eosin. Sections were evaluated by experienced pathologists, blinded to the experimental treatment conditions.

### Enzyme linked immunosorbent assay (ELISA)

Blood from mice, as well as medium supernatant were collected for measuring the concentrations of interleukin- (IL-) 8, monocyte chemotactic protein-1 (MCP-1), IL-1α, IL-6, vascular endothelial growth factor (VEGF) (RayBiotech, GA, US), transforming growth factor-beta (TGF-β), C-reactive protein (CRP), MMP-3, MMP-9 (R&D Systems, MN, US) according to manufacturer’s instructions.

### RT-qPCR

Trizol reagent (Invitrogen, USA) was used to extract total RNA from cells and cDNA was then synthesized using total RNA as template and SuperScript III Reverse Transcriptase system (Thermo Fisher Scientific, USA). PCR was performed using cDNA and SYBR Green Real-Time PCR Master Mixes (Thermo Fisher Scientific, USA) with initial denaturation at 95° C for 1 min, 35 cycles of denaturation at 95° C for 1 min, annealing at 60° C for 2 min, and extension for 30 s, at 72° C. Relative expression levels were calculated using the 2^-ΔΔCt^ method with β-actin and GAPDH as a house keeping gene. Each experiment was repeated independently three times. The primers were listed in Table 2.

**Table 2:** List of the primers used for real-time quantitative PCR.

**Genes**	**Direction**	**Primer sequence**
Human Sirt1 AS	forward	5’-TCTGCTATTACAAGTTACAT-3’
	reverse	5’-CCTGATTATACAGTTCCAA-3’
Mouse Sirt1 AS	forward	5'-AATCCAGTCATTAAACGGTCTACAA-3'
	reverse	5'-TAGGACCATTACTGCCAGAGGA-3’
Sirt1	forward	5’-TTGGCACC GATCCTCGAAC-3’
	reverse	5’-CCCAGCTCCAGTCAGAACTAT-3’
P16	forward	5’-GAACTCTTTCGGTCGTAC-3’
	reverse	5’-CCGTAGTTGAGCAGAAGA-3’
P21	forward	5’- TGATGTCCGACCTGTTCC-3’
	reverse	5’- TGATGTCCGACCTGTTCC-3’
P53	forward	5’- AGTATTTGGATGACAGAA-3’
	reverse	5’- ATGTAGTTGTAGTGGATG-3’
p-selectin	forward	5’- GCCATCCAGAATAAGAATGAA-3’
	reverse	5’- ACCAATCCAGTAGTAAGAGTT-3’
PSGL-1	forward	5’- TCTAACGAGTCTACCATCT-3’
	reverse	5’- AATCAGCCCTTTCTTCAG-3’
vWF	forward	5’- TGGCTCCTCTATGTTGTC-3’
	reverse	5’- TGTGTAACCTCTCCATCAT-3’
Icam1	forward	5’- ACTGGACTATAATCATTC-3’
	reverse	5’- CCTTCTGTAACTTGTATA-3’
Serphine1	forward	5’- CCTTCTGTAACTTGTATA-3’
	reverse	5’- AAGTAGACAGCATTGAGA-3’
β-actin	forward	5’- TATGGAATCCTGTGGCATC-3’
	reverse	5’- GTGTTGGCATAGAGGTCTT-3’

### Western blot assay

Equal concentration of samples was subjected to 15% SDS-PAGE electrophoresis and then transferred to nitrocellulose membranes. The membranes were exposed to appropriate primary antibodies overnight at 4° C, washed and incubated with the appropriate horseradish peroxidase-labeled secondary antibodies for 1 h at room temperature. The bands were detected by chemiluminescence using West Pico Substrate. The primary antibodies against Sirt1(1:20000), P-selectin (anti-CD62P, 1:1000), PSGL-1(1:1000), Von Willebrand factor (1:500), HPI protein (1:2000), FOXO3a (1:1000), Acetylated-Lysine Antibody (1:1000) and second antibody Goat Anti-Rabbit IgG H&L (HRP) were all purchased from Abcam (UK) and Cell signaling technology (MA, USA).

### Co-immunoprecipitation and *in vivo* ubiquitination assay

Senescence HUVECs were transfected with pcDNA vector (lncRNA Sirt1-AS) and/or si-Sirt1. After 48h of transfection, cells were lysed and protein concentration of cellular extracts were determined by Bicinchoninic Acid (BCA) Protein Assay (KeyGen Biotech, China) following the manufacturer's instructions. Cell lysates were incubated with antibodies to FOXO3a and Sirt1 and precipitated with protein A-sapharose (Santa Cruz Biotechnology, CA, USA) at 4° C overnight with constant rotation. After extensively washed with lysis buffer, immunoprecipitants were subjected to electrophoresis through sodium dodecyl sulfate (SDS)-polyacrylamide gel. The separated proteins were then analyzed by western blot assay.

To test the ubiquitination level of FOXO3a, cell lysates were incubated with antibody to FOXO3a and Dynabeads Protein G (Invitrogen, USA) overnight at 4° C. The immunoprecipitated protein was washed and subjected to western blot analysis using anti-Ubiquitin antibody (Cell signaling).

### Statistical analysis

Data were expressed as mean ± Standard Error of Mean (SEM), and analyzed with Graph-Pad Prism 5.0 software (GraphPad Software, San Diego, CA). Statistical analyses were performed using one-way ANOVA, followed by Turkey’s post-test. Differences with *p* < 0.05 were considered statistically significant.
